# Geriatric Hip Fractures and Inpatient Services: Predicting Hospital Charges Using the ASA Score

**DOI:** 10.1155/2014/923717

**Published:** 2014-04-30

**Authors:** Rachel V. Thakore, Young M. Lee, Vasanth Sathiyakumar, William T. Obremskey, Manish K. Sethi

**Affiliations:** The Vanderbilt Orthopaedic Institute Center for Health Policy, Vanderbilt University, Suite 4200, South Tower, MCE, Nashville, TN 37221, USA

## Abstract

*Purpose.* To determine if the American Society of Anesthesiologist (ASA) score can be used to predict hospital charges for inpatient services. *Materials and Methods.* A retrospective chart review was conducted at a level I trauma center on 547 patients over the age of 60 who presented with a hip fracture and required operative fixation. Hospital charges associated with inpatient and postoperative services were organized within six categories of care. Analysis of variance and a linear regression model were performed to compare preoperative ASA scores with charges and inpatient services. *Results.* Inpatient and postoperative charges and services were significantly associated with patients' ASA scores. Patients with an ASA score of 4 had the highest average inpatient charges of services of $15,555, compared to $10,923 for patients with an ASA score of 2. Patients with an ASA score of 4 had an average of 45.3 hospital services compared to 24.1 for patients with a score of 2. *Conclusions.* A patient's ASA score is associated with total and specific hospital charges related to inpatient services. The findings of this study will allow payers to identify the major cost drivers for inpatient services based on a hip fracture patient's preoperative physical status.

## 1. Introduction

Hip fractures, the second leading cause of hospitalization in the elderly [1], cost over $9.8 billion annually in treatment [2]. With the aging US population, it is estimated that over 458,000 to 1,037,000 hip fracture incidents will occur by the year 2050 [3]. As the rate of hip fracture cases increases, so will the costs associated with treating a primarily geriatric patient population that faces longer recovery periods [4], higher risks of opportunistic infections, and prolonged length of stay (LOS) [5–8]. As the Center of Medicare and Medicaid Services (CMS) moves toward a bundled payment model that will reimburse hospitals based on the expected costs of episodes of care, it has become important to understand potential risk factors that are associated with increased inpatient expenses for hip fracture patients. In this context, identifying patient factors that predict resource utilization during inpatient hospitalization can aid in the establishment of a risk-adjusted reimbursement system. One potential risk-adjustment tool that has been considered by the government and other payers is the universally employed American Society of Anesthesiologist (ASA) classification system [9]. Utilized by anesthesiologists to assess a patient's preoperative health status, the ASA classification system assigns patients a score using a five-step scale ranging from normally healthy to moribund [10] (the appendix).

The ASA scoring system has been proven to be a reliable method for predicting LOS and costs associated with geriatric hip fracture patients [6, 11]. Garcia et al. recently demonstrated that a patient's ASA score was a stronger predictor of increased LOS and room and board charges than other well-known predictors of costs such as age, BMI, and comorbidities [6]. However, this study was limited in its scope, since service charges were not analyzed.

Based on the limitations of current literature, the distribution of costs and resources utilized within a patient's hospitalization remains extremely difficult to estimate. A few studies have demonstrated that comprehensive geriatric assessment (CGR)—a multidimensional series of screenings that evaluate a patient's medical, psychosocial, and functional status—may be predictive of surgical outcomes among elderly patients [12–14]. However, CGR is a complex, labor intensive evaluation that may not be feasible for use in all tertiary care centers [15]. A single, ubiquitous tool such as the ASA score that is used in all hospitals may be more widely adopted in predictive models of inpatient expenditure on patients with hip fractures. Therefore, the purpose of the present study was to investigate whether a patient's ASA score is significantly associated with the charges of inpatient services provided during hospitalization of patients with hip fractures.

## 2. Methods

Upon approval from our institutional review board, a retrospective chart review was conducted using our institution's electronic medical records system and the current procedural terminology (CPT) code system. CPT codes describe medical, surgical, and diagnostic services provided by physicians and other medical professionals. The amount of payment provided for services is based on Resource-Based Relative Value Scales (RBRVS), which are determined by calculating the resource costs needed to provide these services. Physician services are identified with the CPT code which is then submitted to insurance companies or Medicare for reimbursement [16].

Our institution is a level I trauma center that practices standard of care treatment where an orthopaedic surgeon is primarily responsible for the care of hip fracture patients. Gerontologists are consulted when needed. Patients were identified as study candidates using CPT code searches for those who had operative management of a hip fracture between January 1, 2000, and December 31, 2009 (CPT codes: 27125, 27236, 27236, 27238, 27244, and 27245). A total of 720 patients were identified using these inclusion criteria. Of these, 170 patients were excluded from analysis due to incomplete medical charts or age exclusion (<60 years). All patients were assigned ASA scores immediately before surgery. One patient with an ASA score of 1 and 2 patients with ASA scores of 5 were also excluded due to low sample sizes in those categories, leaving 547 patients for inclusion in this study.

The remaining 547 patients represented patients who underwent surgical management for an isolated low energy hip fracture who were also over 60 years of age and had complete medical records. The electronic medical charts of these patients were reviewed for information including age, gender, medical comorbidities, ASA score, date of admission, date of operation, date of hospital discharge, and type of operation via CPT code. The ASA score was assigned to each patient by the anesthesiologist before each operation. All inpatient services for these patients during their inpatient stays were obtained from the institution's financial services department. The tests and procedures for each patient were provided in line-item format via CPT code with associated charges for each item. These tests and procedures were subsequently organized into the six broad categories of CPT codes defined by the American Medical Association (AMA): anesthesia, surgery, radiology, evaluation and management, pathology and laboratory, and medicine [17]. Anesthesia (00100–01999 and 99100–99150) included anesthesia administration by an anesthesiologist or nurse anesthetist in a general, regional, or local method. Surgery (10021–69990) included all surgical procedures within ten body systems, including surgical packages and separate procedures. Radiology (70010–79999) included diagnostic imaging and services provided by radiologists and radiology technicians. Evaluation and management (99201–99499) included consultations and services by physicians, nurse practitioners, clinical nurse specialists, certified nurse midwives, and physician assistants. Pathology and laboratory (80047–89398) included clinical laboratory tests related to the blood and lymph. Medicine (90281–99199, 99500–99607) included all medical services and procedures.

17 medical comorbidities were also collected from patient charts utilizing a data management database at our institution. These included a hypertension, myocardial infarction, cardiac dysrhythmia, atrial fibrillation, atrial flutter, congestive heart failure, heart block, cerebrovascular disease, bleeding disorder, chronic obstructive pulmonary disease, emphysema, current smoker, past smoker, renal insufficiency, dialysis dependency, cancer, and diabetes.

Prior to analysis, the normality of the variable distributions was assessed via histogram and Kolmogorov-Smirnov statistic. The charges by CPT code and total number of services provided were not normally distributed. Therefore, the relationship between the preoperative ASA and the total number of services provided during the inpatient stay was assessed using the Kruskal-Wallis nonparametric analysis of variance. Similarly, a Kruskal-Wallis analysis of variance was also performed to identify the relationship between ASA score and total charges of these tests and procedures during the inpatient stay. These analyses were repeated to exclude any inpatient days prior to the day of surgery in order to correlate ASA score to strictly postoperative tests and procedures. The significance of the difference for each of these analyses of variance was evaluated according to a Bonferroni correction at an alpha of 0.05/7 = 0.007 to achieve a family-wise significance rate of 0.05 for each set of analyses. Linear regressions were performed using charges and number of services procedures performed as dependent variables with ASA score as an independent variable, controlled for age, gender, and the 17 comorbidities we collected during our chart review.

## 3. Results

720 patients who underwent hip fracture repair at our institution were found through our search. Patients who were under the age of 60 were excluded from analysis. Patients who had ASA scores of 1 (*n* = 1) or 5 (*n* = 2) were excluded from analysis due to the low sample size in these categories. After applying our exclusion criteria, 547 patients with complete medical records over the age of 60 were included in analysis.  Table 2 provides demographic data for our patient population. The mean age of our patient population was 78 years. The majority of our patients were female (66.4%). Average BMI was within the normal range (24.7).

**Figure 1:**
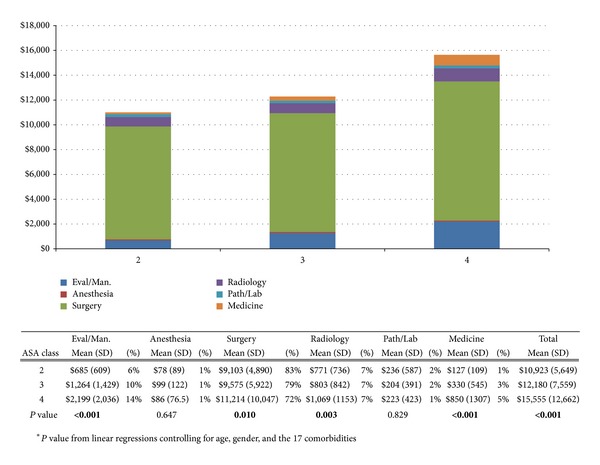
The average charge per patient within six categories of service based on patients' ASA scores. Total average charges are also presented.

Figure 1 shows the average charge within six categories of service in relation to patients' assigned ASA scores. Average charges ranged from $10,923 to $15,555, with surgery comprising the highest proportion of charges for all three ASA classifications. Patients with an ASA score of 4 had the highest average inpatient charges ($15,555), followed by patients with a score of 3 ($12,180). Linear regression models controlled for age, gender, and 17 comorbidities showed that, for every increase in ASA score, there were statistically significant increases in charges for evaluation and management ($799.40, 95% CI $565.70–1033.10 *P* < 0.001), surgery ($1362.30, 95% CI $326.0–$2398.60, *P* = 0.010), radiology ($199.30, 95% CI $70.60–$331.04, *P* = 0.003), and medicine ($389.70, 95% CI $273.80–505.60, *P* < 0.001). There was also an increase in total charges by ASA score ($2751.30, 95% CI $1438.60–$4064.10, *P* < 0.001).

**Figure 2:**
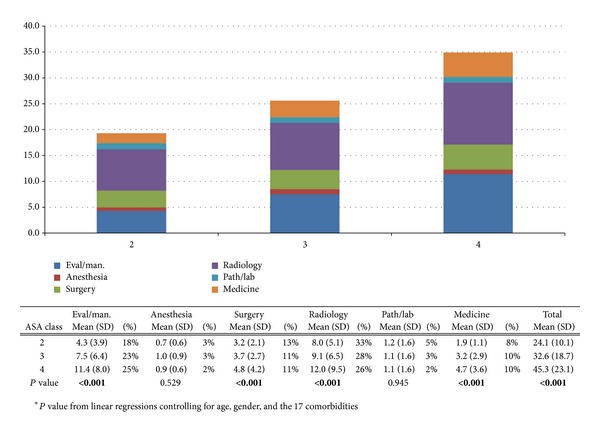
The average frequency of services per patient based on patients' ASA scores. The total average frequency of services is also provided.

An ASA score of 4 was associated with the highest average number of services (45.3) while a score of 2 was associated with the lowest (24.1). Linear regression models controlled for age, gender, and comorbidities showed that, for every increase in ASA score, there were statistically significant increases in number of services for evaluation and management (3.6, 95% CI 2.5–4.6, *P* < 0.001), surgery (1.0, 95% CI 0.5–1.4, *P* < 0.001), radiology (2.4, 95% CI 1.4–3.5, *P* < 0.001), and medicine (1.4, 95% CI 0.9–1.8, *P* < 0.001). There was also an increase in number of services by ASA score overall (11.2, 95% CI 8.3–14.1, *P* < 0.001) (Figure 2).

**Figure 3:**
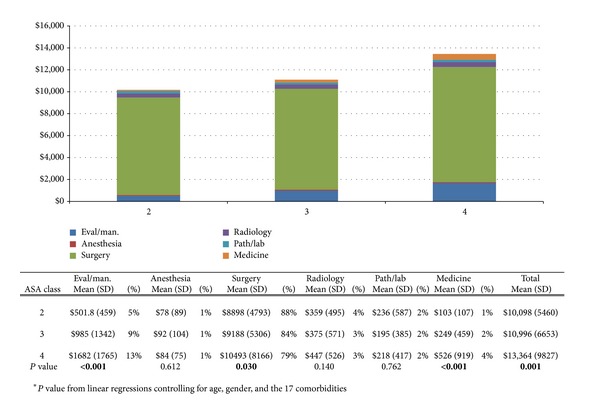
The average charges per patient related to postoperative services based on patients' ASA scores. Total average charges are also presented.

**Figure 4:**
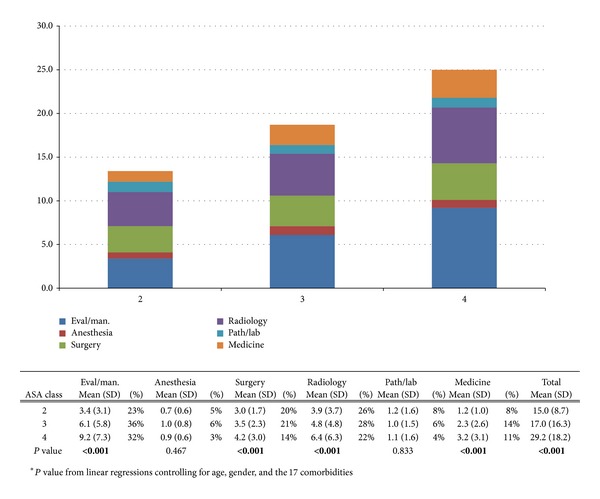
The average frequency of postoperative services per patient based on patients' ASA scores. The total average frequency of services is also provided.

Figures 3 and 4 show the average frequency of postoperative services and associated charges, respectively, based on ASA scores. Patients with an ASA score of 4 had significantly higher average total charges and frequency of services provided for all postoperative tests compared to all other ASA scores. The lowest total charge was associated with an ASA score of 2 ($10,098), followed by scores of 3 ($10,996) and 4 ($13,364). Each increase in ASA score was significantly associated with increases in postoperative evaluation and management charges ($612.40, 95% CI $399.90–824.90, *P* < 0.001), surgical charges ($999.70, 95% CI 95.20–1904.20, *P* = 0.30), medicine charges ($210.60, 95% CI $123.40–297.80, *P* < 0.001), and total charges ($1876.10, 95% CI $763.60–2988.70, *P* = 0.001) (Figure 3). Similarly, an increase in ASA score was also significantly associated with the number of postoperative services provided. Specifically, ASA score was significantly associated with the number of evaluation and management services (2.9, 95% CI 2.0–3.8, *P* < 0.001), number of surgical services (0.7, 95% CI 0.3–1.1, *P* < 0.001), number of radiological services (1.5, 95% CI 0.7–2.3, *P* < 0.001), number of medicine services (0.9, 95% CI 0.5–1.3, *P* < 0.001), and total number of services provided (7.1, 95% CI 4.6–9.6, *P* < 0.001) (Figure 4).

## 4. Discussion

Currently, more than 90% of hip fracture patients over the age of 65 have their hospitalization services covered by Medicare [4]. With the advent of the new bundled payment model, providers are reimbursed based on expected charges for episodes of care [18]. In this new system, reimbursement for treatment will be standardized for all patients, regardless of differences in patient factors that may increase the costs of care. While CGR may be a useful assessment of resource utilization in institutions where orthogeriatric specific management is utilized [19], several factors limit its feasibility in the hospitals where standard of care treatment is provided [15]. The ASA classification system, which is a simple and widely used method of ranking patients based on their preoperative physical status, may be a valuable measure to incorporate in the development of risk-adjusted reimbursement model instead of a global method of payment.

Our investigation demonstrated that ASA scores could be used to predict the number of times inpatient services are provided and the associated charges within six categories of service. Our findings are corroborated by previous studies that have studied the association between ASA scores and hospital charges for hip fracture patients. Garcia et al. recently reported that ASA score was associated with increased LOS which correlated to increased room and board charges at a charge of $4503 per day of hospitalization [6]. In our study, which investigated service charges instead of room and board, we found that the ASA score is a useful method for predicting the frequency and charges of services required for hip fracture patients in our institution and others were similar treatment models that are provided.

The ASA classification was originally developed for use by anesthesiologists to determine risk of operative morbidity [20] based on patients comorbidities. In our study, we have shown that the ASA score can also be used to predict surgical resource utilization. In fact, surgery was disproportionately the most expensive category of service, composing 72%–88% of all charges and only 11%–21% of services provided. Therefore, surgical services may be the best area to focus on quality improvement initiatives in order to most effectively lower costs among hip fracture patients with high ASA scores.

Several reasons explain why surgical charges may increase with ASA score, especially within the postoperative period. The ASA classification system has been shown to be correlated with multiple factors that increase surgical resource utilization including infection [21], reoperations [22], intraoperative blood loss [23], and duration of surgery [24]. Similarly, hip fracture patients with a greater number of comorbidities have been shown to be more likely to suffer postoperative complications that would require diagnostics and imaging [25], which would explain the increase in radiology charges with ASA score.

The number of evaluation and management services and charges also increased significantly with ASA score. One major reason for this is that patients with higher ASA scores are more likely to develop complications. In fact, Donegan et al. recently showed that ASA classification is strongly associated with medical complications that require interventions by a medical specialist or internist after hip fracture surgery [11]. Furthermore, patients with higher ASA scores are likely to have even higher charges than those reported in our study due to repeated readmissions required for complications. In fact, Radcliff et al. reported that a higher ASA score was associated with worse outcomes thirty days after surgery for male hip fracture patients [26]. Therefore, when considering the future costs of readmissions, patients with higher ASA scores would become an even greater financial burden to hospitals and physicians in a global payment system than demonstrated in our study.

Alternatively, patients with higher ASA scores did not require significantly more anesthesia or pathology services during their hospitalization. This may be due to several reasons. Unlike other categories of service such as evaluation and management, services provided by anesthesiologists are not prolonged throughout a patient's duration of hospitalization. Pathology services are not commonly utilized for orthopaedic trauma patients who sustained fracture. In fact, most patients in our study required only one clinical laboratory service regardless of ASA scoring.

### 4.1. Study Limitations

Our study is limited as a retrospective chart review at a single level I trauma center. Although this study design minimized variability in hospital charges and patterns of practice, it also constrains the generalizability of our results to the general patient population and other medical centers. Because very few patients in our analysis were assigned an ASA score of 1 or 5, we could not include these patients in our analysis. We only controlled for 17 comorbidities in our linear regression model. Other medical comorbidities such as dementia and hepatic disease could also correlate to hospital resource utilization. We also did not consider whether medical conditions may have delayed surgical intervention for our patients. Moreover, the ASA classification system is limited by anesthesiologists' subjectivity in assigning scores, although moderate to substantial interrater reliability has been reported when limited to a single surgical specialty [27, 28]. A larger-scale, multi-institutional investigation would strengthen the argument of the use of the ASA score as a tool for predicting inpatient charges and services among the hip fracture population.

## 5. Conclusion

In accordance with past investigations that have demonstrated the predictive power of the ASA classification system in the treatment of hip fracture patients, we have shown that a patient's ASA score is associated with services and charges provided during inpatient hospitalization. Further studies are needed to determine whether the charges associated with ASA score are due to potentially preventable outcomes, such as the development of complications and the need of reoperations, or are necessary expenditure related to severe presentation of injury. Additional studies to look at a day-by-day cost analysis would help identify the association between time and expenditure of resources on patients to help design future interventions to decrease cost. Although the new bundled payment model of reimbursement generalizes utilization of hospital services by episode of care, individualized clinical characteristics of geriatric hip fracture patients, including severity of condition, can have a major influence on hospital expenditure. Because the ASA score is a universally applied method of classification, it should be considered in future reimbursement models in the care of hip fracture patients.

## Figures and Tables

**Table 1:** American Society of Anesthesiology's physical status (ASA PS) class.

ASA score	Definition
1	A normal healthy patient
2	A patient with mild systemic disease
3	A patient with severe systemic disease
4	A patient with severe systemic disease that is a constant threat to life
5	A moribund patient who is not expected to survive without the operation
6	A declared brain-dead patient whose organs are being removed for donor purposes

**Table 2:** Population demographic information.

	*N*	%
Age (yrs)		
60–74	202	13.3%
75+	345	63.1%
Mean	78.0 (9.9)	
Gender		
Male	184	33.6%
Female	363	66.4%
ASA score		
2	58	10.6%
3	371	67.8%
4	118	21.6%
Average BMI (kg/m^2^)	24.7 (6.1)	

## Appendix

See Table 1.

## References

[1] Wilkins K (1999). Health care consequences of falls for seniors. *Health Reports*.

[2] News AAOS Academy Statement on Prevention of Hip Fractures. http://www2.aaos.org.proxy.library.vanderbilt.edu/aaos/archives/acadnews/2000news/c9-17.htm.

[3] Brown CA, Starr AZ, Nunley JA (2012). Analysis of past secular trends of hip fractures and predicted number in the future 2010–2050. *Journal of Orthopaedic Trauma*.

[4] US Congress OoTA (1994). *Hip Fracture Outcomes in People Age 50 and Over—Background Paper*.

[5] Fisher AA, Davis MW, Rubenach SE, Sivakumaran S, Smith PN, Budge MM (2006). Outcomes for older patients with hip fractures: the impact of orthopedic and geriatric medicine cocare. *Journal of Orthopaedic Trauma*.

[6] Garcia AE, Bonnaig JV, Yoneda RE ZT (2012). Patient variables which may predict length of stay and hospital charges in elderly patients with hip fracture. *Journal of Orthopaedic Trauma*.

[7] Long SJ, Brown KF, Ames D, Vincent C (2013). What is known about adverse events in older medical hospital inpatients? A systematic review of the literature. *International Journal for Quality in Health Care*.

[8] Pousada L, Leipzig R, Smyth C, Rosenfeld S, Dooley P, Kennedy RD (1991). Effect of a geriatric consult team on length of acute-care hospital stay for hip fracture. *Einstein Quarterly Journal Of Biology & Medicine*.

[9] Davenport DL, Bowe EA, Henderson WG, Khuri SF, Mentzer RM (2006). National Surgical Quality Improvement Program (NSQIP) risk factors can be used to validate American Society of Anesthesiologists Physical Status Classification (ASA PS) levels. *Annals of Surgery*.

[10] ASA Physical Classification System http://www.asahq.org/clinical/physicalstatus.

[11] Donegan DJ, Gay AN, Baldwin K, Morales EE, Esterhai JL, Mehta S (2010). Use of medical comorbidities to predict complications after hip fracture surgery in the elderly. *The Journal of Bone & Joint Surgery*.

[12] Fukuse T, Satoda N, Hijiya K, Fujinaga T (2005). Importance of a comprehensive geriatric assessment in prediction of complications following thoracic surgery in elderly patients. *Chest*.

[13] Overcash JA, Beckstead J (2008). Predicting falls in older patients using components of a comprehensive geriatric assessment. *Clinical Journal of Oncology Nursing*.

[14] Martinez-Velilla N, Ibanez-eroiz B, Alonso-Renedo J (2013). Is comprehensive geriatric assessment a better 1-year mortality predictor than comorbidity and prognostic indices in hospitalized older adults?. *Journal of the American Geriatrics Society*.

[15] Horgan AM, Leighl NB, Coate L (2012). Impact and feasibility of a comprehensive geriatric assessment in the oncology setting: a pilot study. *American Journal of Clinical Oncology*.

[16] American Medical Association RBRVS: Resource-Based Relative Value Scale. http://www.ama-assn.org//ama/pub/physician-resources/solutions-managing-your-practice/coding-billing-insurance/medicare/the-resource-based-relative-value-scale.page.

[17] Moisio M (2009). *Medical Terminology for Insurance and Coding*.

[18] Centers for Medicare & Medicaid Services Fact Sheets. http://www.cms.gov/apps/media/fact_sheets.asp.

[19] Pioli G, Davoli ML, Pellicciotti F, Pignedoli P, Ferrari A (2011). Comprehensive care. *European Journal of Physical and Rehabilitation Medicine*.

[20] Daabiss M (2011). American Society of Anaesthesiologists physical status classification. *Indian Journal of Anaesthesia*.

[21] Ridgeway S, Wilson J, Charlet A, Katafos G, Pearson A, Coello R (2005). Infection of the surgical site after arthroplasty of the hip. *The Bone & Joint Journal*.

[22] Palm H, Krasheninnikoff M, Holck K (2012). An algorithm for hip fracture surgery reducted the one-year reoperation rate from 18% to 12%. *The Bone & Joint Journal*.

[23] Newman ET, Watters TS, Lewis JS (2014). Impact of perioperative allogeneic and autologous blood transfusion on acute wound infection following total knee and total hip arthroplasty. *The Journal of Bone & Joint Surgery*.

[24] Wolters U, Wolf T, Stützer H, Schröder T (1996). ASA classification and perioperative variables as predictors of postoperative outcome. *British Journal of Anaesthesia*.

[25] Roche JJW, Wenn RT, Sahota O, Moran CG (2005). Effect of comorbidities and postoperative complications on mortality after hip fracture in elderly people: prospective observational cohort study. *British Medical Journal*.

[26] Radcliff TA, Henderson WG, Stoner TJ, Khuri SF, Dohm M, Hutt E (2008). Patient risk factors, operative care, and outcomes among older community-dwelling male veterans with hip fracture. *The Journal of Bone & Joint Surgery*.

[27] Jacqueline R, Malviya S, Burke C, Reynolds P (2006). An assessment of interrater reliability of the ASA physical status classification in pediatric surgical patients. *Paediatric Anaesthesia*.

[28] Ringdal KG, Skaga NO, Steen PA (2013). Classification of comorbidity in trauma: the reliability of pre-injury ASA physical status classification. *Injury*.

